# Supplementary material for: Campylobacter colonization and undernutrition in infants in rural eastern Ethiopia — a longitudinal community-based birth cohort study
Source: Front Public Health. 2025 Jan 7;12:1467462. doi: 10.3389/fpubh.2024.1467462 (PMC11747651; doi:10.3389/fpubh.2024.1467462)
Supplement: Supplementary file 1 [file Table_1.docx]

Supplementary table 1. Determinants of outcomes of *Campylobacter* load and/or decrease in length-for-age Z score.

| Domain |  | Determinant^$^ | Univariate summary^&^ | Effects on outcomes by regression coefficient^$^  (P-value) | | | |
| --- | --- | --- | --- | --- | --- | --- | --- |
|  |  |  |  | *Campylobacte*r load | | Decrease in LAZ^#^ | |
|  |  |  |  | Unadjusted | Adjusted^^^ | Unadjusted | Adjusted^^^ |
| Immediate  causes | Inadequate Dietary intake | Time-varying determinants |  |  |  |  |  |
|  |  | - Achieved MDD^#^ | 165 (29%) | 0.150 (0.439) | -0.124 (0.485) | 0.105 (0.598) | -0.131 (0.518) |
|  | Disease | Baseline determinants |  |  |  |  |  |
|  |  | - WAZ^#^ at enrollment | -0.38 (1.15) | -0.094 (0.060) | -0.087 (0.081) | 0.107 (0.206) | 0.115 (0.175) |
|  |  | - Underweight at enrollment | 12 (11%) | 0.229 (0.200) | 0.211 (0.241) | -0.530 (0.079) | -0.573 (0.058) |
|  |  | Time-varying determinants |  |  |  |  |  |
|  |  | - Vitamin A supplementation^!^ | 52 (5%) | - | - | 4.994 (<0.001) ^*^ | 0.922 (0.227) |
|  |  | - Iron supplementation^!^ | 6 (1%) | - | - | 1.132 (0.204) | 1.798 (0.021) ^*^ |
|  |  | - Treated for malnutrition^!^ | 9 (1%) | - | - | 4.442 (0.038) ^*^ | 0.059 (0.973) |
|  |  | - Vaccinated against rotavirus^!^ | 70 (14%) | - | - | 1.623 (<0.001) ^*^ | 0.631 (0.087) |
|  |  | - Current diarrhea^!^ | 191 (19%) | - | - | 1.516 (<0.001) ^*^ | 0.338 (0.240) |
|  |  | - Current fever^!^ | 147 (14%) | - | - | 1.304 (<0.001) ^*^ | 0.154 (0.628) |
|  |  | - Any vaccination^!^ | 160 (16%) | - | - | 2.010 (<0.001) ^*^ | -0.052 (0.881) |
|  |  | - Vaccinated against measles^!^ | 9 (2%) | - | - | 2.962 (0.090) | 0.176 (0.898) |
|  |  | - Vaccinated against polio^!^ | 97 (9%) | - | - | 2.034 (<0.001)^*^ | -0.293 (0.500) |

Supplementary table 1. Continued

| Domain |  | Determinant^$^ | Univariate summary^&^ | Effects on outcomes by regression coefficient^$^  (P-value) | | | |
| --- | --- | --- | --- | --- | --- | --- | --- |
|  |  |  |  | *Campylobacte*r load | | Decrease in LAZ^#^ | |
|  |  |  |  | Unadjusted | Adjusted^^^ | Unadjusted | Adjusted^^^ |
| Immediate  causes | Disease | *Campylobacter* load (unit: log_10_[gene copies per 50 ng of DNA])^!^ | 2.91 (0.58) |  |  |  |  |
|  |  | - Effect on decrease in LAZ^#^ |  | - | - | 0.194 (0.245) | 0.165 (0.320) |
|  |  | - longitudinal effect on LAZ |  | - | - | 0.543 (<0.001) ^*^ | 0.01 (0.897) |
| Underlying  causes | Household food security | Time-varying determinants |  |  |  |  |  |
|  |  | - HFIAS^#^ | 4.73 (5.39) | 0.066 (<0.001) ^*^ | 0.040 (<0.001) ^*^ | 0.124 (<0.001) ^*^ | 0.061 (<0.001) ^*^ |
|  |  | - Food Secure (i.e., HFIAS = 1) | 361 (35%) | -0.939  (<0.001) ^*^ | -0.319 (0.015) ^*^ | -1.156 (<0.001) ^*^ | -0.337 (0.074) |
|  | Inadequate care and feeding practices | Baseline determinants |  |  |  |  |  |
|  |  | - Colostrum feeding | 100 (95%) | -0.581 (0.216) | -0.493 (0.284) | 0.219 (0.678) | 0.274 (0.607) |
|  |  | - Early initiation of breastfeeding | 71 (68%) | -0.326 (0.150) | -0.342 (0.118) | -0.162 (0.499) | -0.167 (0.487) |
|  |  | - Pre-lacteal feeding | 66 (67%) | 0.505 (0.030) ^*^ | 0.505 (0.027) ^*^ | 0.064 (0.799) | 0.043 (0.864) |
|  |  | Age/duration of feeding practices |  |  |  |  |  |
|  |  | - Age at first introduction of complementary feeding (days) | 186.60 (45.90) | <0.001 (0.709) | <0.001 (0.743) | -0.001 (0.715) | -0.001 (0.670) |

Supplementary table 1. Continued

| Domain |  | Determinant^$^ | Univariate summary^&^ | Effects on outcomes by regression coefficient^$^  (P-value) | | | |
| --- | --- | --- | --- | --- | --- | --- | --- |
|  |  |  |  | *Campylobacte*r load | | Decrease in LAZ^#^ | |
|  |  |  |  | Unadjusted | Adjusted^^^ | Unadjusted | Adjusted^^^ |
| Underlying  causes | Inadequate care and feeding practices | Time-varying determinants |  |  |  |  |  |
|  |  | - Current breastfeeding | 1029 (100%) | 1.896 (0.194) | 0.729 (0.513) | 1.640 (0.111) | 0.210 (0.800) |
|  |  | - Received (semi) solid foods | 137 (13%) | 1.559 (<0.001) ^*^ | 0.393(0.044) ^*^ | 3.409 (<0.001) ^*^ | 0.083 (0.867) |
|  |  | - Treated with ORS^#!^ | 89 (9%) | - | - | 1.987 (<0.001) ^*^ | 0.205 (0.659) |
|  |  | - Taken to a health center when experiencing diarrhea^!^ | 174 (17%) | - | - | 2.019 (<0.001) ^*^ | -0.092 (0.789) |
|  |  | - Taken to a health center when experiencing fever^!^ | 177 (17%) | - | - | 2.209 (<0.001) ^*^ | 0.349 (0.294) |
|  | Unhealthy household environment and inadequate health services^$^ | Baseline determinants |  |  |  |  |  |
|  |  | - Prenatal care when pregnant^!^ | 80 (76%) | - | - | 0.436 (0.054) ^*^ | 0.448 (0.048) ^*^ |
|  |  | - Prenatal care location^!^ |  |  |  |  |  |
|  |  | Health post | 42 (55%) | - | - | -0.379 (0.274) | -0.313 (0.375) |
|  |  | Health clinic | 24 (31%) | - | - | -0.051 (0.891) | 0.030 (0.937) |
|  |  | Government run hospitals | 11 (14%) | - | - | -^%^ | -^%^ |
|  |  | - Access to drinking water^%^ | 52 (56%) | -0.324 (0.166) | -0.359 (0.129) | -0.090 (0.821) | -0.043 (0.915) |
|  |  | Safely managed | 1 (1%) | - | - | - | - |
|  |  | Basic | 51 (55%) | - | - | - | - |
|  |  | Limited | 34 (37%) | - | - | - | - |
|  |  | Unimproved | 7 (8%) | - | - | - | - |
|  |  | Surface water | 0 | - | - | - | - |

Supplementary table 1. Continued

| Domain |  | Determinant^$^ | Univariate summary^&^ | Effects on outcomes by regression coefficient^$^  (P-value) | | | |
| --- | --- | --- | --- | --- | --- | --- | --- |
|  |  |  |  | *Campylobacte*r load | | Decrease in LAZ^#^ | |
|  |  |  |  | Unadjusted | Adjusted^^^ | Unadjusted | Adjusted^^^ |
| Underlying  causes | Unhealthy household environment and inadequate health services^$^ | - Access to any sanitation^%^ |  | -0.113 (0.384) | -0.107 (0.420) | 0.178 (0.428) | 0.253 (0.263) |
|  |  | Safely managed | 0 | - | - | - | - |
|  |  | Basic | 9 (9%) | - | - | - | - |
|  |  | Limited | 2 (2%) | - | - | - | - |
|  |  | Unimproved | 18 (18%) | - | - | - | - |
|  |  | Open defecation | 69 (70%) | - | - | - | - |
|  |  | - Access to any hygiene facilities^%^ |  | -0.055 (0.687) | -0.079 (0.571) | -0.109 (0.644) | -0.098 (0.679) |
|  |  | Basic | 20 (21%) | - | - | - | - |
|  |  | Limited | 5 (5%) | - | - | - | - |
|  |  | No facility | 72 (74%) | - | - | - | - |
|  |  | - Handwashing after going to the toilet/latrine | 106 (100%) | - | - | - | - |
|  |  | - Handwashing after cleaning a baby's bottom/changing a baby's nappy | 89 (84%) | -0.002 (0.988) | 0.009 (0.955) | -0.069 (0.793) | -0.017 (0.949) |
|  |  | - Handwashing before preparing/handling food | 98 (92%) | 0.175 (0.413) | 0.181 (0.399) | -0.302 (0.408) | -0.266 (0.465) |
|  |  | - Handwashing before feeding an infant/eating | 88 (83%) | -0.035 (0.818) | -0.040 (0.799) | -0.010 (0.968) | 0.074 (0.778) |
|  |  | - Handwashing after handling raw food | 46 (44%) | -0.040 (0.725) | -0.029 (0.800) | -0.042 (0.829) | -0.008 (0.966) |
|  |  | - Handwashing after handling garbage | 80 (75%) | -0.218 (0.095) | -0.214 (0.102) | 0.083 (0.712) | 0.097 (0.665) |
|  |  | - Handwashing after field work | 86 (82%) | -0.292 (0.046) ^*^ | -0.273 (0.065) | 0.038 (0.881) | 0.058 (0.821) |
|  |  | - Handwashing after handling livestock | 62 (58%) | -0.067 (0.557) | -0.080 (0.489) | -0.198 (0.310) | -0.187 (0.341) |

Supplementary table 1. Continued

| Domain |  | Determinant^$^ | Univariate summary^&^ | Effects on outcomes by regression coefficient^$^  (P-value) | | | |
| --- | --- | --- | --- | --- | --- | --- | --- |
|  |  |  |  | *Campylobacte*r load | | Decrease in LAZ^#^ | |
|  |  |  |  | Unadjusted | Adjusted^^^ | Unadjusted | Adjusted^^^ |
| Underlying  causes | Unhealthy household environment and inadequate health services^$^ | - Handwashing with |  |  |  |  |  |
|  |  | Soap and water | 25 (24%) | -0.223 (0.093) | -0.255 (0.060) | -0.031 (0.892) | -0.019 (0.936) |
|  |  | Water | 80 (76%) |  |  |  |  |
|  |  | - Mother using soap to wash hands | 34 (32%) | -0.311 (0.009) ^*^ | -0.315 (0.008) ^*^ | -0.231 (0.262) | -0.221 (0.283) |
|  |  | - Cleaning infants, post-defecation | 80 (75%) | 0.027 (0.838) | 0.032 (0.809) | -0.012 (0.957) | 0.016 (0.944) |
|  |  | Handwashing after cleaning infants | 69 (86%) | -0.028 (0.886) | 0.019 (0.928) | 0.499 (0.129) | 0.596 (0.082) |
|  |  | - Disposal of infant’s stool, post-defecation^%^ |  |  |  |  |  |
|  |  | Left in the open | 3 (3%) | 0.589 (0.215) | 0.754 (0.129) | 1.030 (0.207) | 0.898 (0.294) |
|  |  | Buried | 15 (14%) | 0.113 (0.758) | 0.214 (0.570) | 0.347 (0.582) | 0.253 (0.698) |
|  |  | Thrown into garbage | 34 (32%) | 0.200 (0.567) | 0.253 (0.472) | 0.486 (0.418) | 0.415 (0.496) |
|  |  | Put/rinsed into drain or ditch | 37 (35%) | 0.074 (0.832) | 0.095 (0.787) | 0.606 (0.312) | 0.491 (0.421) |
|  |  | Put/rinsed into toilet or latrine | 14 (13%) | -0.109 (0.768) | -0.033 (0.930) | 0.198 (0.754) | 0.145 (0.822) |
|  |  | Child used toilet/latrine | 3 (3%) | -^%^ | -^%^ | -^%^ | -^%^ |
|  |  | Time-varying determinants |  |  |  |  |  |
|  |  | - Putting soil/animal feces in mouth | 387 (38%) | 1.563 (<0.001) ^*^ | 0.483 (0.003) ^*^ | 2.207 (<0.001) ^*^ | 0.402 (0.214) |
|  |  | - Preventing putting soil/animal feces in mouth | 489 (51%) | 1.458 (<0.001) ^*^ | 0.108 (0.513) | 1.821  (<0.001) ^*^ | 0.176 (0.508) |
|  |  | - Drinking from a bottle with nipple | 399 (39%) | 1.191 (<0.001) ^*^ | -0.165 (0.280) | 2.148 (<0.001) ^*^ | -0.173 (0.578) |

Supplementary table 1. Continued

| Domain |  | Determinant^$^ | Univariate summary^&^ | Effects on outcomes by regression coefficient^$^  (P-value) | | | |
| --- | --- | --- | --- | --- | --- | --- | --- |
|  |  |  |  | *Campylobacte*r load | | Decrease in LAZ^#^ | |
|  |  |  |  | Unadjusted | Adjusted^^^ | Unadjusted | Adjusted^^^ |
| Control measures | - | Time-varying determinants |  |  |  |  |  |
|  |  | - Any antibiotic use | 185 (18%) | 0.625 (0.003) ^*^ | -0.166 (0.330) | 2.607 (<0.001) ^*^ | 0.253 (0.482) |
| Risks | Exposure to enteric pathogens^@^ | Baseline determinants |  |  |  |  |  |
|  |  | - Cattle count | 0.86 (1.18) | 0.020 (0.689) | 0.022 (0.660) | 0.098 (0.240) | 0.126 (0.136) |
|  |  | - Chicken count | 2.11 (3.42) | -0.008 (0.648) | -0.010 (0.551) | 0.018 (0.535) | 0.016 (0.573) |
|  |  | - Goat count | 1.46 (1.80) | -0.008 (0.812) | -0.008 (0.793) | -0.034 (0.524) | -0.028 (0.597) |
|  |  | - Sheep count | 1.10 (1.82) | -0.030 (0.342) | -0.031 (0.331) | 0.004 (0.943) | 0.012 (0.822) |
|  |  | - Chicken daytime location risk score^%^ |  |  |  |  |  |
|  |  | 2 | 43 (41%) | 0.174 (0.133) | 0.158 (0.183) | -0.377 (0.054) | -0.381 (0.057) |
|  |  | 1 | 2 (2%) | 0.436 (0.294) | 0.427 (0.305) | -1.077 (0.125) | -1.080 (0.123) |
|  |  | 0 | 61 (58%) | -^%^ | -^%^ | -^%^ | -^%^ |
|  |  | - Chicken nighttime location risk score^%^ |  |  |  |  |  |
|  |  | 2 | 43 (41%) | 0.154 (0.197) | 0.142 (0.247) | -0.321 (0.115) | -0.315 (0.128) |
|  |  | 1 | 10 (9%) | 0.107 (0.595) | 0.078 (0.708) | -0.320 (0.347) | -0.319 (0.363) |
|  |  | 0 | 53 (50%) | -^%^ | -^%^ | -^%^ | -^%^ |

Supplementary table 1. Continued

| Domain |  | Determinant^$^ | Univariate summary^&^ | Effects on outcomes by regression coefficient^$^  (P-value) | | | |
| --- | --- | --- | --- | --- | --- | --- | --- |
|  |  |  |  | *Campylobacte*r load | | Decrease in LAZ^#^ | |
|  |  |  |  | Unadjusted | Adjusted^^^ | Unadjusted | Adjusted^^^ |
| Risks | Exposure to enteric pathogens^@^ | - Cattle daytime location risk score^%^ |  |  |  |  |  |
|  |  | 2 | 0 | - | - | - | - |
|  |  | 1 | 26 (25%) | 0.185 (0.156) | 0.199 (0.127) | 0.033 (0.882) | 0.038 (0.867) |
|  |  | 0 | 79 (75%) | -^%^ | -^%^ | -^%^ | -^%^ |
|  |  | - Cattle nighttime location risk score^%^ |  |  |  |  |  |
|  |  | 2 | 0 | - | - | - | - |
|  |  | 1 | 50 (48%) | 0.155 (0.172) | 0.167 (0.145) | 0.241 (0.214) | 0.282 (0.147) |
|  |  | 0 | 55 (52%) | -^%^ | -^%^ | -^%^ | -^%^ |
|  |  | - Sheep daytime location risk score^%^ |  |  |  |  |  |
|  |  | 2 | 0 | - | - | - | - |
|  |  | 1 | 20 (19%) | -0.156 (0.279) | -0.155 (0.286) | -0.282 (0.251) | -0.330 (0.182) |
|  |  | 0 | 86 (81%) | -^%^ | -^%^ | -^%^ | -^%^ |
|  |  | - Sheep nighttime location risk score^%^ |  |  |  |  |  |
|  |  | 2 | 2 (2%) | 0.044 (0.915) | -0.049 (0.908) | 0.041 (0.954) | 0.125 (0.865) |
|  |  | 1 | 45 (42%) | -0.219 (0.057) | -0.225 (0.052) | 0.009 (0.963) | 0.041 (0.835) |
|  |  | 0 | 59 (56%) | -^%^ | -^%^ | -^%^ | -^%^ |
|  |  | - Goat daytime location risk score^%^ |  |  |  |  |  |
|  |  | 2 | 0 | - | - | - | - |
|  |  | 1 | 36 (34%) | -0.091 (0.445) | -0.076 (0.525) | -0.252 (0.214) | -0.225 (0.268) |
|  |  | 0 | 70 (66%) | -^%^ | -^%^ | -^%^ | -^%^ |

Supplementary table 1. Continued

| Domain |  | Determinant^$^ | Univariate summary^&^ | Effects on outcomes by regression coefficient^$^  (P-value) | | | |
| --- | --- | --- | --- | --- | --- | --- | --- |
|  |  |  |  | *Campylobacte*r load | | Decrease in LAZ^#^ | |
|  |  |  |  | Unadjusted | Adjusted^^^ | Unadjusted | Adjusted^^^ |
| Risks | Exposure to enteric pathogens^@^ | - Goat nighttime location risk score^%^ |  |  |  |  |  |
|  |  | 2 | 0 | - | - | - | - |
|  |  | 1 | 58 (55%) | 0.141 (0.214) | 0.127 (0.268) | 0.088 (0.649) | 0.099 (0.612) |
|  |  | 0 | 48 (45%) | -^%^ | -^%^ | -^%^ | -^%^ |
|  |  | - Collection of livestock waste | 94 (89%) | 0.045 (0.803) | 0.024 (0.895) | 0.063 (0.835) | 0.117 (0.707) |
|  |  | - Livestock dropping present in household/homestead | 63 (59%) | 0.167 (0.145) | 0.163 (0.156) | 0.283 (0.148) | 0.270 (0.167) |
|  |  | - Slaughtering owned livestock | 47 (44%) | -0.191 (0.092) | -0.222 (0.053) | 0.151 (0.437) | 0.140 (0.475) |
|  |  | Handwashing related to slaughtering |  |  |  |  |  |
|  |  | Before and after | 30 (64%) | -0.055 (0.773) | -0.024 (0.900) | 0.108 (0.744) | 0.086 (0.800) |
|  |  | After | 17 (36%) | -^%^ | -^%^ | -^%^ | -^%^ |
|  |  | Time-varying determinants |  |  |  |  |  |
|  |  | - Raw milk consumption | 85 (8%) | 1.811 (<0.001) ^*^ | 0.693 (0.006) ^*^ | 3.912 (<0.001) ^*^ | 0.821 (0.129) |
|  |  | - Crawling where animal droppings might be present | 376 (38%) | 1.537 (<0.001) ^*^ | 0.342 (0.051) | 2.235 (<0.001) ^*^ | 0.365 (0.289) |
|  |  | - Physical contact with livestock | 429 (42%) | 1.461 (<0.001) ^*^ | 0.292 (0.055) | 1.970 (<0.001) ^*^ | 0.187 (0.496) |

Supplementary table 1. Continued

| Domain |  | Determinant^$^ | Univariate summary^&^ | Effects on outcomes by regression coefficient^$^  (P-value) | | | |
| --- | --- | --- | --- | --- | --- | --- | --- |
|  |  |  |  | *Campylobacte*r load | | Decrease in LAZ^#^ | |
|  |  |  |  | Unadjusted | Adjusted^^^ | Unadjusted | Adjusted^^^ |
| Benefits | Food production | Time-varying determinant |  |  |  |  |  |
|  |  | - ASF^#^ consumption | 369 (65%) | -0.111 (0.509) | -0.181 (0.223) | 0.164 (0.357) | 0.123 (0.479) |
|  | Livestock income | Baseline determinants |  |  |  |  |  |
|  |  | - Total annual income quartile from selling livestock | - |  |  |  |  |
|  |  | Q4 | - | 0.052 (0.744) | 0.015 (0.926) | 0.042 (0.880) | 0.060 (0.831) |
|  |  | Q3 | - | 0.105 (0.506) | 0.114 (0.492) | -0.015 (0.956) | 0.111 (0.700) |
|  |  | Q2 | - | 0.320 (0.043) ^*^ | 0.302 (0.059) | -0.044 (0.872) | -0.022 (0.936) |
|  |  | Q1 | - | -^%^ | -^%^ | -^%^ | -^%^ |
|  |  | - TLU^#^ | 0.86 (0.92) | 0.003 (0.957) | 0.007 (0.918) | 0.097 (0.356) | 0.141 (0.192) |
| Basic causes | Social/cultural | Baseline determinants |  |  |  |  |  |
|  |  | - Muslim as Mother’s religion | 105 (100%) | - | - | - | - |
|  |  | - Mother’s involvement in khat production | 18 (19%) | -0.136 (0.377) | -0.125 (0.419) | -0.413 (0.118) | -0.413 (0.117) |
|  | Economics/livelihood | Baseline determinants |  |  |  |  |  |
|  |  | - On-farm as primary livelihood | 97 (92%) | 0.462 (0.030) ^*^ | 0.423 (0.053) | 0.263 (0.473) | 0.245 (0.511) |
|  |  | - Asset quartile | - | -0.019 (0.867) | - | -0.274 (0.154) | - |

Supplementary table 1. Continued

| Domain |  | Determinant^$^ | Univariate summary^&^ | Effects on outcomes by regression coefficient^$^  (P-value) | | | |
| --- | --- | --- | --- | --- | --- | --- | --- |
|  |  |  |  | *Campylobacte*r load | | Decrease in LAZ^#^ | |
|  |  |  |  | Unadjusted | Adjusted^^^ | Unadjusted | Adjusted^^^ |
| Basic causes | Human capital | Baseline determinants |  |  |  |  |  |
|  |  | - Mother’s age (years) | 27.08 (6.32) | -0.010 (0.292) | -0.007 (0.446) | -0.006 (0.694) | -0.001 (0.959) |
|  |  | - Mother attended school | 29 (28%) | -0.002 (0.986) | -0.050 (0.706) | -0.275 (0.204) | -0.317 (0.159) |
|  | Basic demographics | Baseline determinants |  |  |  |  |  |
|  |  | - Infant being female | 51 (48%) | 0.151 (0.179) | - | 0.157 (0.416) | - |
|  |  | Time-varying determinants |  |  |  |  |  |
|  |  | - Infant’s age |  |  |  |  |  |
|  |  | Grp^#^ 5 vs. Grp 1 | - | - | - | 1.520 (<0.001) ^*^ | - |
|  |  | Grp(Q^#^) 4 vs. Grp(Q) 1 | - | 1.835 (<0.001) ^*^ | - | 1.356 (<0.001) ^*^ | - |
|  |  | Grp(Q) 3 vs. Grp(Q) 1 | - | 1.400 (<0.001) ^*^ | - | 1.125 (<0.001) ^*^ | - |
|  |  | Grp(Q) 2 vs. Grp(Q) 1 | - | 0.499 (<0.001) ^*^ | - | 0.637 (<0.001) ^*^ | - |

^!^ Determinants for growth faltering but not for *Campylobacter* colonization.

^@^There are conceptual overlaps between *unhealthy household environment* and livestock-related *risks of exposure to enteric pathogens*, determinants more related to smallholder livestock production were categorized into the latter, given this work’s specific focus.

^#^Abbreviations: MDD: minimum dietary diversity, WAZ: weight-for-age Z score, HFIAS: household food insecurity access score, ORS: oral rehydration solution, ASF: animal-sourced foods, TLU: tropical livestock unit. Grp: group, Q: quartile. LAZ: length-for-age Z score.

^$^For baseline determinants, infant feeding indicators shortly after birth were associated with *Campylobacter* load in month one of age and the first available LAZ measure; other determinants were associated with the average *Campylobacter* load of the whole follow-up period and LAZ difference between the first and last available visits. For time-varying (longitudinal) determinants, the averaged *Campylobacter* load of each age quartile was regressed on the concurrent proportion/average of each determinant; the LAZ of each measuring age group was regressed on the corresponding preceding effect of each determinant – the preceding effect was defined as a proportion for categorical variables, or an average for the HFIAS score or the *Campylobacter* load.

^%^Reference group: for binary determinants, the status of absence was treated as the reference; WaSH ladders were binarized in regressions and the categories of unimproved, open defecation, and no facility were respectively treated as reference groups for the *drinking water*, *sanitation*, and *hygiene* ladders, and other categories of each WaSH ladders were merged into a non-reference category for regressions; For *disposal of infant’s stool, post-defecation*, we combined the categories of *Left in the open*, *Buried*, *Thrown into garbage* as the non-reference group, and the other three categories as the reference group in the multiple-determinant model. Definition of each category of livestock location risk score – 0: household did not have the livestock species or kept them outside the house; 1: household kept the livestock species inside the house and confined them; 2: household kept the livestock species inside the house but did not confine them.

^^^Adjusted for sex and socioeconomic status (indicated by a binary variable of household asset) at baseline, and age was further adjusted through random intercepts in linear-mixed models for time-varying determinants.

^&^For categorical variables, frequency (percentage) is presented; for continuous variables, mean (standard deviation) is presented.

^*^p<0.05.

Supplementary table 2. Determinants of environmental enteric dysfunction.

| Domain |  | Determinant^$^ | Effects by regression coefficient^$^  (P-value) | |
| --- | --- | --- | --- | --- |
|  |  |  | Unadjusted | Adjusted^^^ |
| Immediate  causes | Inadequate Dietary intake | Time-varying determinants |  |  |
|  |  | - Achieved MDD^#^ | 1.272 (0.129) | 1.694 (0.060) |
|  | Disease | Baseline determinants |  |  |
|  |  | - WAZ^#^ at enrollment | 0.220 (0.227) | 0.214 (0.255) |
|  |  | - Underweight at enrollment | -0.443 (0.492) | -0.389 (0.559) |
|  |  | Time-varying determinants |  |  |
|  |  | - Vitamin A supplementation^!^ | 6.048 (0.055) | 7.492 (0.019) ^*^ |
|  |  | - Iron supplementation^!^ | 5.467 (0.477) | 3.330 (0.662) |
|  |  | - Treated for malnutrition^!^ | 0.700 (0.896) | -0.369 (0.947) |
|  |  | - Vaccinated against rotavirus^!^ | -0.211 (0.862) | -0.297 (0.810) |
|  |  | - Current fever^!^ | 4.821 (0.010) ^*^ | 4.838 (0.010) ^*^ |
|  |  | - Any vaccination^!^ | -1.124 (0.451) | -0.998 (0.512) |
|  |  | - Vaccinated against measles^!^ | -3.022 (0.424) | -2.736 (0.479) |
|  |  | - Vaccinated against polio^!^ | -1.464 (0.456) | -1.837 (0.365) |
|  |  | - *Campylobacter* load^!^ | 0.128 (0.715) | 0.120 (0.740) |

Supplementary table 2. Continued

| Domain |  | Determinant^$^ | Effects by regression coefficient^$^  (P-value) | |
| --- | --- | --- | --- | --- |
|  |  |  | Unadjusted | Adjusted^^^ |
| Underlying  causes | Household food security | Time-varying determinants |  |  |
|  |  | - HFIAS^#^ | 0.045 (0.448) | 0.033 (0.601) |
|  |  | - Food Secure (i.e., HFIAS = 1) | -1.575 (0.105) | -1.649 (0.100) |
|  | Inadequate care and feeding practices | Baseline determinants |  |  |
|  |  | - Colostrum feeding | 0.238 (0.816) | 0.044 (0.966) |
|  |  | - Early initiation of breastfeeding | -0.143 (0.745) | -0.122 (0.785) |
|  |  | - Pre-lacteal feeding | -0.307 (0.497) | -0.220 (0.638) |
|  |  | Age/duration of feeding practices |  |  |
|  |  | - Age at first introduction of complementary feeding (days) | 0.003 (0.426) | 0.003 (0.501) |

Supplementary table 2. Continued

| Domain |  | Determinant^$^ | Effects by regression coefficient^$^  (P-value) | |
| --- | --- | --- | --- | --- |
|  |  |  | Unadjusted | Adjusted^^^ |
| Underlying  causes | Inadequate care and feeding practices | Time-varying determinants |  |  |
|  |  | - Current breastfeeding | -171.842 (0.992) | -169.454 (0.992) |
|  |  | - Received (semi) solid foods | -1.651 (0.372) | -1.920 (0.308) |
|  |  | - Treated with ORS^#!^ | 0.709 (0.732) | 0.285 (0.894) |
|  |  | - Taken to a health center when experiencing diarrhea^!^ | 2.087 (0.221) | 2.165 (0.215) |
|  |  | - Taken to a health center when experiencing fever^!^ | 1.096 (0.480) | 1.184 (0.456) |
|  | Unhealthy household environment and inadequate health services^$^ | Baseline determinants |  |  |
|  |  | - Prenatal care when pregnant^!^ | 0.304 (0.520) | 0.321 (0.501) |
|  |  | - Prenatal care location^!^ |  |  |
|  |  | Health post | -2.408 (0.028) ^*^ | -2.645 (0.024) ^*^ |
|  |  | Health clinic | -2.207 (0.052) | -2.310 (0.051) |
|  |  | Government run hospitals | -^%^ | -^%^ |
|  |  | - Access to safely managed or basic drinking water^%^ | 0.150 (0.859) | 0.294 (0.740) |
|  |  | Safely managed | - | - |
|  |  | Basic | - | - |
|  |  | Limited | - | - |
|  |  | Unimproved | - | - |
|  |  | Surface water | - | - |

Supplementary table 2. Continue

| Domain |  | Determinant^$^ | Effects by regression coefficient^$^  (P-value) | |
| --- | --- | --- | --- | --- |
|  |  |  | Unadjusted | Adjusted^^^ |
| Underlying  causes | Unhealthy household environment and inadequate health services^$^ | - Access to any sanitation^%^ | 0.468 (0.320) | 0.791 (0.125) |
|  |  | Safely managed | - | - |
|  |  | Basic | - | - |
|  |  | Limited | - | - |
|  |  | Unimproved | - | - |
|  |  | Open defecation | - | - |
|  |  | - Access to any hygiene facilities^%^ | -0.418 (0.389) | -0.403 (0.423) |
|  |  | Basic | - | - |
|  |  | Limited | - | - |
|  |  | No facility | - | - |
|  |  | - Handwashing after going to the toilet/latrine | - | - |
|  |  | - Handwashing after cleaning a baby's bottom/changing a baby's nappy | -0.028 (0.959) | 0.082 (0.883) |
|  |  | - Handwashing before preparing/handling food | 0.243 (0.773) | 0.456 (0.604) |
|  |  | - Handwashing before feeding an infant/eating | 0.886 (0.116) | 1.198 (0.050) |
|  |  | - Handwashing after handling raw food | -0.109 (0.792) | -0.022 (0.959) |
|  |  | - Handwashing after handling garbage | 0.527 (0.266) | 0.535 (0.268) |
|  |  | - Handwashing after field work | -1.153 (0.061) | -1.260 (0.047) |
|  |  | - Handwashing after handling livestock | -0.476 (0.258) | -0.503 (0.240) |

Supplementary table 2. Continued

| Domain |  | Determinant^$^ | Effects by regression coefficient^$^  (P-value) | |
| --- | --- | --- | --- | --- |
|  |  |  | Unadjusted | Adjusted^^^ |
| Underlying  causes | Unhealthy household environment and inadequate health services^$^ | - Handwashing with |  |  |
|  |  | Soap and water | 0.015 (0.976) | -0.037 (0.941) |
|  |  | Water |  |  |
|  |  | - Mother using soap to wash hands | 0.035 (0.936) | -0.033 (0.941) |
|  |  | - Cleaning infants, post-defecation | -0.682 (0.183) | -0.691 (0.183) |
|  |  | Handwashing after cleaning infants | -0.120 (0.855) | -0.074 (0.919) |
|  |  | - Disposal of infant’s stool, post-defecation |  |  |
|  |  | Left in the open | 0.693 (0.711) | 0.114 (0.953) |
|  |  | Buried | 0.000 (1.000) | -0.580 (0.715) |
|  |  | Thrown into garbage | 0.348 (0.812) | 0.095 (0.949) |
|  |  | Put/rinsed into drain or ditch | 0.057 (0.969) | -0.427 (0.775) |
|  |  | Put/rinsed into toilet or latrine | 0.588 (0.699) | 0.384 (0.804) |
|  |  | Child used toilet/latrine | -^%^ | -^%^ |
|  |  | - Putting soil/animal feces in mouth | 1.663 (0.195) | 1.700 (0.194) |
|  |  | - Preventing putting soil/animal feces in mouth | 1.843 (0.227) | 2.207 (0.170) |
|  |  | - Drinking from a bottle with nipple | -1.847 (0.152) | -1.594 (0.226) |

Supplementary table 2. Continued

| Domain |  | Determinant^$^ | Effects by regression coefficient^$^  (P-value) | |
| --- | --- | --- | --- | --- |
|  |  |  | Unadjusted | Adjusted^^^ |
| Control measures |  | - Any antibiotic use | 0.443 (0.774) | 0.720 (0.652) |
| Risks | Exposure to enteric pathogens^@^ | Baseline determinants |  |  |
|  |  | - Cattle count | 0.061 (0.765) | 0.115 (0.581) |
|  |  | - Chicken count | 0.112 (0.122) | 0.111 (0.150) |
|  |  | - Goats count | 0.021 (0.848) | 0.025 (0.828) |
|  |  | - Sheep count | -0.205 (0.123) | -0.210 (0.111) |
|  |  | - Chicken daytime location risk score^%^ |  |  |
|  |  | 2 | 0.236 (0.572) | 0.285 (0.512) |
|  |  | 1 | 15.457 (0.992) | 15.009 (0.992) |
|  |  | 0 | -^%^ | -^%^ |
|  |  | - Chicken nighttime location risk score^%^ |  |  |
|  |  | 2 | 0.104 (0.808) | 0.191 (0.670) |
|  |  | 1 | 2.037 (0.064) | 2.203 (0.054) |
|  |  | 0 | -^%^ | -^%^ |

Supplementary table 2. Continued

| Domain |  | Determinant^$^ | Effects by regression coefficient^$^  (P-value) | |
| --- | --- | --- | --- | --- |
|  |  |  | Unadjusted | Adjusted^^^ |
| Risks | Exposure to enteric pathogens^@^ | - Cattle daytime location risk score^%^ |  |  |
|  |  | 2 | - | - |
|  |  | 1 | -0.081 (0.864) | -0.083 (0.862) |
|  |  | 0 | -^%^ | -^%^ |
|  |  | - Cattle nighttime location risk score^%^ |  |  |
|  |  | 2 | - | - |
|  |  | 1 | 0.102 (0.804) | 0.165 (0.695) |
|  |  | 0 | -^%^ | -^%^ |
|  |  | - Sheep daytime location risk score^%^ |  |  |
|  |  | 2 | - | - |
|  |  | 1 | -0.006 (0.991) | -0.033 (0.952) |
|  |  | 0 | -^%^ | -^%^ |
|  |  | - Sheep nighttime location risk score^%^ |  |  |
|  |  | 2 | -15.789 (0.991) | -15.611 (0.991) |
|  |  | 1 | 0.065 (0.876) | 0.089 (0.835) |
|  |  | 0 | -^%^ | -^%^ |
|  |  | - Goat daytime location risk score^%^ |  |  |
|  |  | 2 | - | - |
|  |  | 1 | 0.117 (0.786) | 0.160 (0.716) |
|  |  | 0 | -^%^ | -^%^ |

Supplementary table 2. Continued

| Domain |  | Determinant^$^ | Effects by regression coefficient^$^  (P-value) | |
| --- | --- | --- | --- | --- |
|  |  |  | Unadjusted | Adjusted^^^ |
| Risks | Exposure to enteric pathogens^@^ | - Goat nighttime location risk score^%^ |  |  |
|  |  | 2 | - | - |
|  |  | 1 | 0.083 (0.839) | 0.071 (0.868) |
|  |  | 0 | -^%^ | -^%^ |
|  |  | - Collection of livestock waste | -0.686 (0.343) | -0.748 (0.322) |
|  |  | - Livestock dropping present in household/homestead | 0.377 (0.368) | 0.410 (0.343) |
|  |  | - Slaughtering owned livestock | -0.499 (0.228) | -0.598 (0.166) |
|  |  | Handwashing related to slaughtering | -0.277 (0.666) | -0.342 (0.612) |
|  |  | Before and after |  |  |
|  |  | After | -^%^ | -^%^ |
|  |  | Time-varying determinants |  |  |
|  |  | - Raw milk consumption | -0.339 (0.853) | -0.139 (0.940) |
|  |  | - Crawling where animal droppings might be present | 1.295 (0.298) | 1.379 (0.279) |
|  |  | - Physical contact with livestock | 1.553 (0.118) | 1.963 (0.065) |

Supplementary table 2. Continued

| Domain |  | Determinant^$^ | Effects by regression coefficient^$^  (P-value) | |
| --- | --- | --- | --- | --- |
|  |  |  | Unadjusted | Adjusted^^^ |
| Benefits | Food production | Time-varying determinant |  |  |
|  |  | - ASF^#^ consumption | -0.692 (0.405) | -0.510 (0.553) |
|  | Livestock income | Baseline determinants |  |  |
|  |  | - Total annual income quartile from selling livestock |  |  |
|  |  | Q4 | -0.167 (0.773) | -0.073 (0.907) |
|  |  | Q3 | 0.169 (0.771) | 0.437 (0.492) |
|  |  | Q2 | 0.238 (0.680) | 0.405 (0.509) |
|  |  | Q1 | -^%^ | -^%^ |
|  |  | - TLU^#^ | 0.004 (0.987) | 0.064 (0.808) |
| Basic causes | Social/cultural | Baseline determinants |  |  |
|  |  | - Muslim as Mother’s religion | - | - |
|  |  | - Mother’s involvement in khat production | -0.280 (0.597) | -0.381 (0.494) |
|  | Economics/livelihood | Baseline determinants |  |  |
|  |  | - On-farm as primary livelihood | 0.274 (0.710) | 0.300 (0.694) |
|  |  | - Asset quartile | -0.438 (0.287) | - |

Supplementary table 2. Continued

| Domain |  | Determinant^$^ | Effects by regression coefficient^$^  (P-value) | |
| --- | --- | --- | --- | --- |
|  |  |  | Unadjusted | Adjusted^^^ |
| Basic causes | Human capital | Baseline determinants |  |  |
|  |  | - Mother’s age (years) | 0.009 (0.774) | 0.022 (0.508) |
|  |  | - Mother attended school | -0.101 (0.827) | -0.155 (0.748) |
|  | Basic demographics | Baseline determinants |  |  |
|  |  | - Infant being female | 0.254 (0.537) | - |
|  |  | Time-varying determinants |  |  |
|  |  | - Infant’s age at EED sampling | 0.006 (0.278) |  |

^@^There are conceptual overlaps between *unhealthy household environment* and livestock-related *risks of exposure to enteric pathogens*, determinants more related to smallholder livestock production were categorized into the latter, given this work’s specific focus.

^#^Abbreviations: EED: environmental enteric dysfunction, MDD: minimum dietary diversity, WAZ: weight-for-age Z score, HFIAS: household food insecurity access score, ORS: oral rehydration solution, ASF: animal-sourced foods, TLU: tropical livestock unit. 0: household did not have the livestock species or kept them outside the house; 1: household kept the livestock species inside the house and confined them; 2: household kept the livestock species inside the house but did not confine them. Q: quartile.

^$^We associated the EED outcome on the overall effect (i.e., proportion or the average of HFIAS scores) of the whole follow-up of each time-varying (longitudinal) determinants.

^%^Reference group: for binary determinants, the status of absence was treated as the reference; WaSH ladders were binarized in regressions and the categories of unimproved, open defecation, and no facility were respectively treated as reference groups for the *drinking water*, *sanitation*, and *hygiene* ladders, and other categories of each WaSH ladders were merged into a non-reference category for regressions. Definition of each category of livestock location risk score – 0: household did not have the livestock species or kept them outside the house; 1: household kept the livestock species inside the house and confined them; 2: household kept the livestock species inside the house but did not confine them.

^^^Adjusted for sex and socioeconomic status (indicated by a binary variable of household asset) at baseline, and age at EED sampling.

^*^p<0.05.
